# Unraveling the Complexity of Soil Microbiomes in a Large-Scale Study Subjected to Different Agricultural Management in Styria

**DOI:** 10.3389/fmicb.2020.01052

**Published:** 2020-05-25

**Authors:** Martina Köberl, Philipp Wagner, Henry Müller, Robert Matzer, Hans Unterfrauner, Tomislav Cernava, Gabriele Berg

**Affiliations:** ^1^Institute of Environmental Biotechnology, Graz University of Technology, Graz, Austria; ^2^ARGE obst.wein, Association of Weinbauverband Steiermark and Verband Steirischer Erwerbsobstbauern, Graz, Austria; ^3^TB Unterfrauner GmbH, Vienna, Austria

**Keywords:** soil microbiome, vineyards, orchards, edaphic parameters, herbicide usage

## Abstract

Healthy soil microbiomes are crucial for achieving high productivity in combination with crop quality, but our understanding of microbial diversity is still limited. In a large-scale study including 116 composite samples from vineyards, orchards and other crops from all over Styria (south-east Austria), agricultural management as well as distinct soil parameters were identified as drivers of the indigenous microbial communities in agricultural soils. The analysis of the soil microbiota based on microbial profiling of prokaryotic 16S rRNA gene fragments and fungal ITS regions revealed high bacterial and fungal diversity within Styrian agricultural soils; 206,596 prokaryotic and 53,710 fungal OTUs. Vineyards revealed a significantly higher diversity and distinct composition of soil fungi over orchards and other agricultural soils, whereas the prokaryotic diversity was unaffected. Soil pH was identified as one of the most important edaphic modulators of microbial community structure in both, vineyard and orchard soils. In general, the acid-base balance, disorders in the soil sorption complex, content and quality of organic substance as well as individual nutrients were identified as important drivers of the microbial community structure of Styrian vineyard and orchard soils. However, responses to distinct parameters differed in orchards and vineyards, and prokaryotic and fungal community responded differently to the same abiotic factor. In comparison to orchards, the microbiome of vineyard soils maintained a higher stability when herbicides were applied. Orchard soils exhibited drastic shifts within community composition; herbicides seem to have a substantial impact on the bacterial order *Chthoniobacterales* as well as potential plant growth promoters and antagonists of phytopathogens (*Flavobacterium*, *Monographella*), with a decreased abundance in herbicide-treated soils. Moreover, soils of herbicide-treated orchards revealed a significantly higher presence of potential apple pathogenic fungi (*Nectria*, *Thelonectria*). These findings provide the basis to adapt soil management practices in the future in order to maintain a healthy microbiome in agricultural soils.

## Introduction

Soil is a non-renewable natural bio-resource involved in important ecosystem functions and biogeochemical cycles on earth. One of the most important characteristics of a soil ecosystem is soil health, which is the result of biotic as well as abiotic processes and connected to various interactions within the system. These interactions have strong impact on the microbial activity, supporting many central processes in soil (Fra̧c et al., 2018). The cycling of carbon and other nutrients or the promotion of plant growth are found among a broad variety of functions ascribed to soil microorganisms (Jansson and Hofmockel, 2018). Soil health is also fundamental for food security and safety as well as carbon storage (FAO and ITPS, 2015). Microbial communities as well as other organisms which reside in soils are extremely complex and diverse. Millions of species and billions of individual organisms can be found in various soils ranging from microorganisms such as bacteria, archaea, fungi, and protists to larger organism like ants and earthworms. Moreover, 1,000s of individual taxa including members of all three domains of life can live in one gram of soil (Fierer, 2017). Bacterial species form the biggest group by number and also by diversity (Gagelidze et al., 2018). Biotic and abiotic factors, including soil pH, temperature, soil type, geographic and climatic conditions, shape the microbiome of bulk and rhizospheric soil (Santoyo et al., 2017). Plant species influence soil microbial diversity (Berg and Smalla, 2009) and *vice versa* (Bardgett and van der Putten, 2014). Soils are characterized by a high degree of spatial structuring; they are composed of micro-aggregates (< 0.25 mm), which bind soil organic carbon and protect it from removal by erosion, and of macro-aggregates (0.25 to 2 mm), which limit oxygen diffusion and regulate water flow; each of the aggregates provides a unique ecological niche with its characteristic microbiome structure (Wilpiszeski et al., 2019). In fact, it has been suggested that soils are the ecosystems with the most diverse composition of microbiota on earth as a consequence of so many different niches being present at small spatial scales (Jansson, 2011; Prosser, 2015). However, this microbial biodiversity is largely understudied, and regional or global overviews are rare. The first global atlas of soil bacterial taxa revealed a region-specific composition (Delgado-Baquerizo et al., 2018), but regional studies are barely available, e.g., for Austria.

Styria is a region in south-east Austria and characterized by a uniquely high diversity of soil types. Together with the Mediterranean-influenced climate, the hilly area provides excellent conditions for cultivation of a large variety of grape and pome (mainly apple) cultivars, including the indigenous ‘Schilcher’ grape and ‘Kronprinz Rudolf’ apple. Both crops are therefore of economic importance for the Styrian agriculture but also world-wide (FAOSTAT, 2016, 2017; International Organisation of Vine and Wine, 2016). Compared to international standards, Austria has a small-scaled agriculture. Austria counted in 2015 a total of 14,133 viticulture holdings, of which 2,085 holdings were located in Styria. The total area of Styrian grapevine (*Vitis vinifera* L.) production is 4,329.22 hectares in size, which also includes non-productive vineyards. Most of this area (3,337.37 hectares) is attributed to the production of white wine, while the remaining area is used for red wine production. Apple (*Malus domestica* Borkh.) is due to its climatic adaption the most planted tree fruit of the temperate zone and one of the most cultivated in the world (Ahmed et al., 2011; Eccher et al., 2014). Austria’s apple production covers a total area of 7,700 hectares, managed by 3,909 commercial fruit producers. The largest part of this area is located in the state of Styria with 5,900 hectares, which corresponds to 77% of Austria’s total apple production area. The quantity of harvested apples in Styria is close to 100,000 tons per year. In 2017, the Styrian area for organic apple production amounted to 1,195 hectares, corresponding to 20% (all data for Austria/Styria were obtained from STATISTIK AUSTRIA^1^).

A recent study on the microbiome of vineyard soils in the Italian province of Trentino highlighted the need to characterize bacterial and fungal communities of the soil microbiota to fully understand the factors that drive their variability. It was found that while the bacterial component of the microbiome had a core of conserved species that accounted for more than 60% of the reads of each sample, and that was shaped both by location and land use, the core fungal microbiome was smaller and determined by geographic factors that dominated differences due to land management (Coller et al., 2019). The rhizospheric microbiome of grapevines is also strongly influenced by host genetic control, namely by the rootstock genotype. This was recently confirmed for vineyards in Spain (Berlanas et al., 2019) and Italy (Marasco et al., 2018). Interestingly, despite these host-related differences in the taxonomic structure of the microbiome, Marasco et al. (2018) observed a homeostatic effect on the distribution of plant growth-promoting abilities. The impact of the rootstock genotype on the microbiome structure has been reported for apple trees as well (Liu et al., 2018). However, a greater number of significant effects in apple orchards were observed due to different soil management practices such as soil amendment with *Brassica* seed meal (Mazzola et al., 2015) or varying manure ratios (Zhang et al., 2013).

The focus of the present study was the detailed assessment of the soil microbiome of vineyards and orchards in Styria/Austria subjected to different agricultural practices. Altogether, 116 soil samples were collected in a large-scale approach and analyzed in terms of their prokaryotic and fungal diversity and community composition. Complementary cultivation-independent and statistical analyses were performed to identify prevalent taxa and characterize the soil microbiome. In order to determine the impact of abiotic soil parameters, physico-chemical parameters were ascertained in detail by fractional analysis, separating water-soluble, exchangeable, and subsequently deliverable (reserve) fractions. Microbiome shifts resulting from the usage of herbicides were investigated based on the treatment history of each cultivation site.

## Materials and Methods

### Sampling and Metagenomic DNA Extraction

Soil samples were collected in early spring (March/April) 2017 in nine different regions of Styria in Austria: Feldbach (6 samples), Gleisdorf (24), Hartberg (16), Kitzeck (9), Leibnitz (8), Leutschach (23), Südoststeiermark (14), Südsteiermark (5), and Weststeiermark (11). Altogether, 116 composite soil samples consisting of five sub-samples each were collected in a horizon of 10–30 cm depth, which are assigned to three general sample groups: vineyards (73 samples), orchards (32; 28 apple orchards, 3 pear orchards, and 1 quince orchard), and other agricultural soils (11) of diverse usage (grassland, soy bean, rye, oat).

In order to isolate total community DNA, 5 g of soil were mixed with 15 ml of sterile 0.85% NaCl and placed for 10 min on a shaking platform. Subsequently, 3 ml of the suspensions were centrifuged (20 min, 16,000 × *g*, 4°C) and the resulting pellets were stored at −70°C until further processing. This approach enables the usage of a greater amount of input soil material comprising micro- and macro-aggregates. DNA was extracted using the FastDNA SPIN Kit for Soil (MP Biomedicals, Solon, OH, United States) and quantified using a NanoDrop 2000c spectrophotometer (Thermo Scientific, Waltham, MA, United States).

### Edaphic Parameters and Herbicide Usage

Physico-chemical soil parameters were assessed through fractional analysis according to ÖNORM S 2122-1 by TB Unterfrauner (Vienna, Austria). This analysis separates water-soluble, exchangeable, and subsequently deliverable (reserve) fraction by considering the different bond types of elements in soil impacting their accessibility for plants. Water-soluble elements define the concentrations of substances in the soil solution, the most important source for plant nutrition. Organic and mineral parts in soil build the sorption complex; their negatively charged surfaces adsorb cations, which can be exchanged to become available for roots, e.g., through root excretion or fertilization – those elements are referred to as exchangeable. Water-soluble and exchangeable elements are considered as plant-available, whereas the subsequently deliverable (reserve) fraction will become accessible to plants by natural weathering processes within 10 to 15 years. Soil pH was determined in water (soil–water saturation extract) as well as in neutral salt solution (1M KCl). Soil texture (KH), electrical conductivity (EC) and water-soluble elements were analyzed based on soil–water saturation extracts. Exchangeable elements were assessed from LiCl extracts and the subsequently deliverable elements (reserve fraction) from HCl extracts. Total contents of C, N and S were determined by dry combustion. Lime content was calculated as CaCO_3_ using a Scheibler calcimeter. More details about the fractional analysis can be found under https://www.bodenoekologie.com/en/. Information about treatment history was gathered from the farmers by a questionnaire.

### PCR-Based Barcoding

Microbial profiling was performed according to the standards of the Earth Microbiome Project^2^. The hypervariable V4–V5 region of the 16S rRNA gene was amplified with the primers 515F-Y/926R (Quince et al., 2011; Parada et al., 2016), which carried sequence pads for later extension with sample specific tags. The reaction mixture for the first PCR (10 μl) contained 1 × Taq-&GO (MP Biomedicals, Solon, OH, United States), 0.1 μM of each primer, and 1 μl of template DNA (95°C, 3 min; 35 cycles of 95°C, 45 s; 55°C, 45 s; 72°C, 90 s; and elongation at 72°C, 5 min). Individual golay_12 barcodes (Caporaso et al., 2012) were attached in a second PCR (30 μl) comprising 1 × Taq-&GO, 0.2 μM of each primer, and 1 μl of the first PCR mixtures (95°C, 5 min; 15 cycles of 95°C, 30 s; 53°C, 30 s; 72°C, 30 s; and elongation at 72°C, 5 min). For the fungal community, the ITS1 region was amplified with the primer pair ITS1f/ITS2 (White et al., 1990; Gardes and Bruns, 1993) carrying the sequence pads for later golay_12 barcode extension. The first PCR (10 μl) consisted of 1 × Taq-&GO, 3 mM MgCl_2_, 0.1 μM of each primer, and 1 μl of template DNA (94°C, 5 min; 30 cycles of 94°C, 30 s; 58°C, 35 s; 72°C, 40 s; and elongation at 72°C, 10 min). The second PCR (30 μl) comprised 1 × Taq-&GO, 0.2 μM of each primer, and 1.8 μl of the first PCR mixtures (95°C, 5 min; 15 cycles of 95°C, 30 s; 53°C, 30 s; 72°C, 30 s; and elongation at 72°C, 5 min). For the prokaryotic and for the fungal community, PCR products of three independent reactions were pooled in equal volumes and purified by employing the Wizard SV Gel and PCR Clean-Up System (Promega, Madison, WI, United States). Paired-end Illumina HiSeq sequencing (2 × 300 bp) was conducted by GATC Biotech (Konstanz, Germany).

### Data Analysis

Data analysis was performed by employing QIIME 1.9.1 (Caporaso et al., 2010a). Joined paired-end reads with more than three consecutive low-quality base calls (Phred quality score ≤ 25) were truncated at the position where their quality began to drop, and only reads with > 75% consecutive high-quality base calls, without any ambiguous characters, and longer than 200 nucleotides in length were retained for further analyses. Demultiplexed high-quality 16S rRNA gene fragments were *de novo* clustered into operational taxonomic units (OTUs) with uclust (Edgar, 2010), using a 97% similarity threshold. For each OTU, the most abundant sequence was selected as representative, and the taxonomy was assigned with the uclust-based consensus taxonomy assigner against the Greengenes database (version 13.8). The representative sequence set was aligned with PyNAST (Caporaso et al., 2010b), and potential chimeric sequences were discarded based on a check with ChimeraSlayer. Joined, quality-filtered and demultiplexed ITS reads were cleaned from chimeras using the usearch7 algorithm and open-reference picked into OTUs with uclust against the dynamic UNITE database (version 7.1). The taxonomy of the representative ITS sequences was assigned with blast against the same reference database. OTU tables were constructed and singletons, doubletons, and reads for which taxonomy could not be assigned were removed from the datasets. The prokaryotic dataset was further filtered to remove cyanobacterial and mitochondrial sequences, and fungal dataset was filtered to remove bacterial and archaeal reads. When joining paired-end reads from the ITS regions, there is a bias against species with long ITS regions which exceed the sequencing length and are therefore discarded during the joining process (Hoggard et al., 2018). We examined this effect by comparing the results of merging read pairs to analysis using only the forward reads (Supplementary Table 1).

For alpha and beta diversity analyses, OTU tables were rarefied to the lowest number of reads per sample (all samples: 1,847 reads for 16S rRNA genes and 7,992 reads for ITS; herbicide impact: 10,653 reads for 16S rRNA genes and 74,662 reads for ITS). For metadata-based comparisons, only soil samples from vineyards and orchards were considered. The impact of herbicide usage was analyzed on a subset of samples with known treatment history (5 samples per treatment for vineyards and orchards, respectively). Organic sites have been under this management since at least two years. Alpha diversity was evaluated based on Shannon, Chao1 and the observed_otus metric; significant differences were calculated using the non-parametric two-sample *t*-test with 999 Monte Carlo permutations. Beta diversity was analyzed based on Bray–Curtis dissimilarities; ANOSIM and adonis tests with 999 permutations were used for corresponding statistics. Significances for differences in the abundances of taxa were determined based on the Bonferroni-corrected Kruskal–Wallis test with 1,000 permutations. Differences in OTU abundances between herbicide-treated and organic sites were assessed with the non-parametric *t*-test with 1,000 Monte Carlo permutations. Heat maps were visualized in Heatmapper (Babicki et al., 2016). Fungal communities were classified according their predicted trophic mode using FUNGuild (Nguyen et al., 2016); significant differences between herbicide-treated and organic sites were calculated with SPSS Statistics 26 (SPSS, Inc., Chicago, IL, United States) using the independent samples *t*-test. PICRUSt (version 1.1.1; Langille et al., 2013) analyses were performed to predict metabolic functions of the prokaryotic communities. Closed-reference picked (Greengenes version 13.8) rarefied OTU tables were used as input material, and the KO (KEGG orthology) database served as reference database. Predicted metagenome tables were analyzed with STAMP (Statistical Analysis of Metagenomic Profiles; version 2.1.3; Parks et al., 2014), the two-sided Welch’s *t*-test was used for statistical comparisons.

## Results

### The Soil Microbiome of Styrian Vineyards and Orchards

The analysis of the soil microbiome based on high-throughput amplicon sequencing of 16S rRNA gene fragments and the fungal internal transcribed spacer (ITS) 1 region revealed high bacterial and fungal diversity within Styrian agricultural soils. Overall, 206,596 prokaryotic OTUs were identified with a total of 10,808,936 reads. The fungal dataset revealed 53,710 OTUs and a total read count of 23,927,374.

**FIGURE 1:**
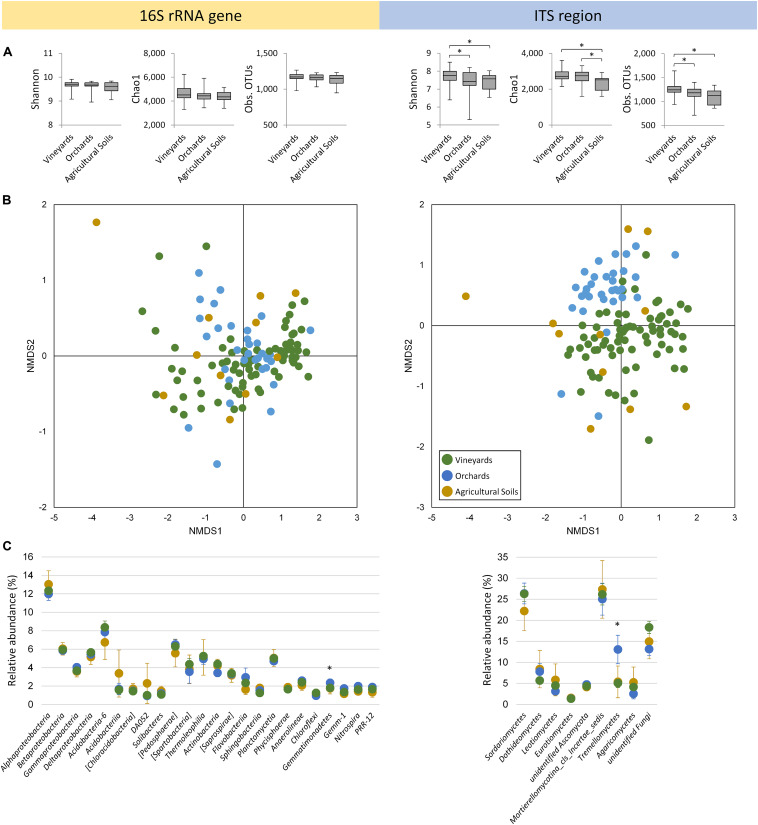
Microbial soil communities colonizing vineyards, orchards, and other agricultural soils in Styria/Austria. **(A)** Alpha diversity determined by Shannon, Chao1 index, and observed OTUs in the rarefied datasets. Significant differences (non-parametric two-sample *t*-test, *p* ≤ 0.05) are indicated by asterisks. **(B)** Non-metric multidimensional scaling (NMDS) plots based on Bray–Curtis dissimilarities. The corresponding 2D stress values are 0.1 and 0.2, respectively for 16S rRNA genes and ITS regions. **(C)** Taxonomic composition visualizing classes with > 1% of overall relative abundance. Data are mean values ± confidences; asterisks indicate significant differences (Bonferroni-corrected Kruskal–Wallis test, *p* ≤ 0.05) in relation to soil usage.

Alpha diversity indicated significant differences (*p* ≤ 0.05) in relation to soil usage (vineyards, orchards, and other agricultural soils) for the fungal diversity, whereas the prokaryotic diversity was unaffected by this factor (Figure 1A). Fungal diversity was detected to be highest in vineyard soils. Non-metric multidimensional scaling (NMDS) analyses based on Bray–Curtis dissimilarity matrices revealed a clustering of vineyard and orchard samples for the fungal community (Figure 1B; *R* = 0.26, *p* = 0.001). The prokaryotic community showed no significant separation of soil samples originating from vineyards and orchards (*R* = 0.02, *p* = 0.263). Soil usage was responsible for only 1.7% (*p* = 0.006) of prokaryotic and 6.0% (*p* = 0.001) of fungal community variation between vineyards or orchards. The sample group with other agricultural soils revealed broad scattering in both datasets, which reflects different agricultural usage (grassland, soy bean, rye, oat). One outlier within agricultural soil samples, which clearly separated from all the others in the prokaryotic and in the fungal dataset, originated from a recently cleared woodland currently under green manuring.

*Proteobacteria* were identified as the most dominant bacterial phylum in vineyards, orchards, and other agricultural soils, encompassing 27.7, 27.4, and 27.8%, respectively. At the class level, they could be affiliated to *Alpha*-, *Beta*-, *Gamma-*, and *Delta proteobacteria* (Figure 1C), whereby *Alphaproteobacteria* were the most prevalent (vineyards 12.3%, orchards 12.0%, and other agricultural soils 13.0%). Besides *Proteobacteria*, *Acidobacteria* (classes *Acidobacteria*-6, *Acidobacteriia*, [*Chloracidobacteria*], DA052 and *Solibacteres*), *Verrucomicrobia* ([*Pedosphaerae*] and [*Spartobacteria*]), *Actinobacteria* (*Thermoleophilia* and *Actinobacteria*), *Bacteroidetes* ([*Saprospirae*], *Flavobacteriia* and *Sphingobacteriia*), *Planctomycetes* (*Planctomycetia* and *Phycisphaerae*), and *Chloroflexi* (*Anaerolineae* and *Chloroflexi*) were found as dominant soil inhabitants. Additionally, *Gemmatimonadetes* (*Gemmatimonadetes* and Gemm-1), *Nitrospirae* (*Nitrospira*), and WS3 (PRR-12) were found in an overall relative abundance over 1%. The fungal soil community was dominated by *Ascomycota* (vineyards 43.8%, orchards 45.0%, and other agricultural soils 44.8%), which could be divided into *Sordariomycetes*, *Dothideomycetes*, *Leotiomycetes*, *Eurotiomycetes* and unidentified *Ascomycota*. High abundances were also observed for *Zygomycota* (*Mortierellomycotina*_cls_*Incertae_sedis*), *Basidiomycota* (*Tremellomycetes* and *Agaricomycetes*) and unidentified *Fungi*. Significant differences (*p* ≤ 0.05) in relation to soil usage were observed for the bacterial class *Gemmatimonadetes* (also for the phylum *Gemmatimonadetes*) and the fungal class *Tremellomycetes*, both with highest abundances in orchard soils.

### Impact of Edaphic Parameters on Soil Microbiomes

**FIGURE 2:**
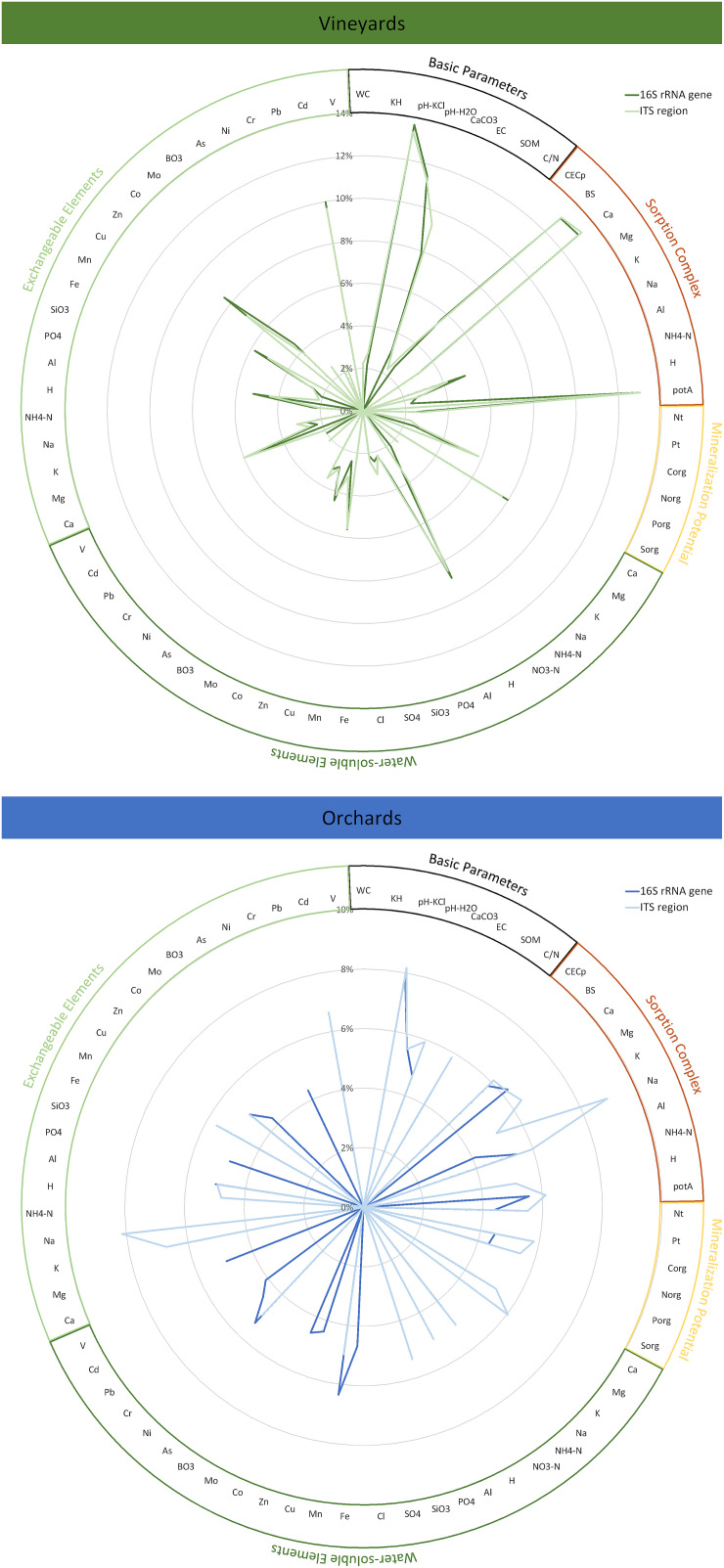
Variance within the prokaryotic (16S rRNA gene) and fungal (ITS region) community inhabiting Styrian vineyard and orchard soils explained by the individual edaphic parameters. Significant variations (*p* ≤ 0.05) were determined with adonis based on Bray–Curtis dissimilarities. Abiotic soil parameters were ascertained in detail by fractional analysis, whereby the currently not available reserve fraction was not considered in the analysis of community impact. Basic parameters: WC = water content, KH = soil texture, pH-KCl = pH in neutral KCl solution, pH-H_2_O = pH in water, CaCO_3_ = lime content, EC = electrical conductivity, SOM = soil organic matter, C/N = C/N ratio of SOM; Sorption complex: CECp = potential cation exchange capacity, BS = base saturation, substance ratios in the sorption complex (Ca, Mg, K, Na, Al, NH_4_-N, H, potA = potential acids); Mineralization potential: Nt, Pt = total content of N and P, Corg, Norg, Porg, Sorg = organic pool of C, N, P, S; Water-soluble elements: Ca, Mg, K, Na, NH_4_-N, NO_3_-N, H, Al, PO_4_, SiO_3_, SO_4_, Cl, Fe, Mn, Cu, Zn, Co, Mo, BO_3_, As, Ni, Cr, Pb, Cd, V; Exchangeable elements: Ca, Mg, K, Na, NH_4_-N, H, Al, PO_4_, SiO_3_, Fe, Mn, Cu, Zn, Co, Mo, BO_3_, As, Ni, Cr, Pb, Cd, V.

Abiotic soil parameters were ascertained in detail by fractional analysis, separating water-soluble, exchangeable, and subsequently deliverable (reserve) fractions. For vineyard soils, the parameters mostly affecting prokaryotic and fungal community variation are highly similar, while orchard soils revealed a generally higher complexity within their community drivers with divergent importance for prokaryotes and fungi (Figure 2). The parameter with the most significant influence on all microbial communities was the soil pH (vineyards: 14% for prokaryotes, 13% for fungi; orchards: 8% for prokaryotes, 8% for fungi). Vineyard communities were also affected by calcium concentrations – within all fractions, but especially in the sorption complex (13% for both communities) – and consequently base saturation (13% for both communities). Potential acids in the sorption complex also substantially influenced community variation in vineyard soils (13% for both communities). The proportions of variance explained by those parameters were in general higher for the vineyard than for the orchard microbiomes. The prokaryotic orchard communities were in addition to soil pH (8%) and calcium in the sorption complex (6%) most significantly affected by water-soluble manganese (6%), whereas the fungal variation within orchard soils was in addition to soil pH (8%) mostly driven by sodium concentrations – sorption complex (9%) and exchangeable fraction (8%).

### Impact of Herbicide Usage on Soil Microbiomes

**FIGURE 3:**
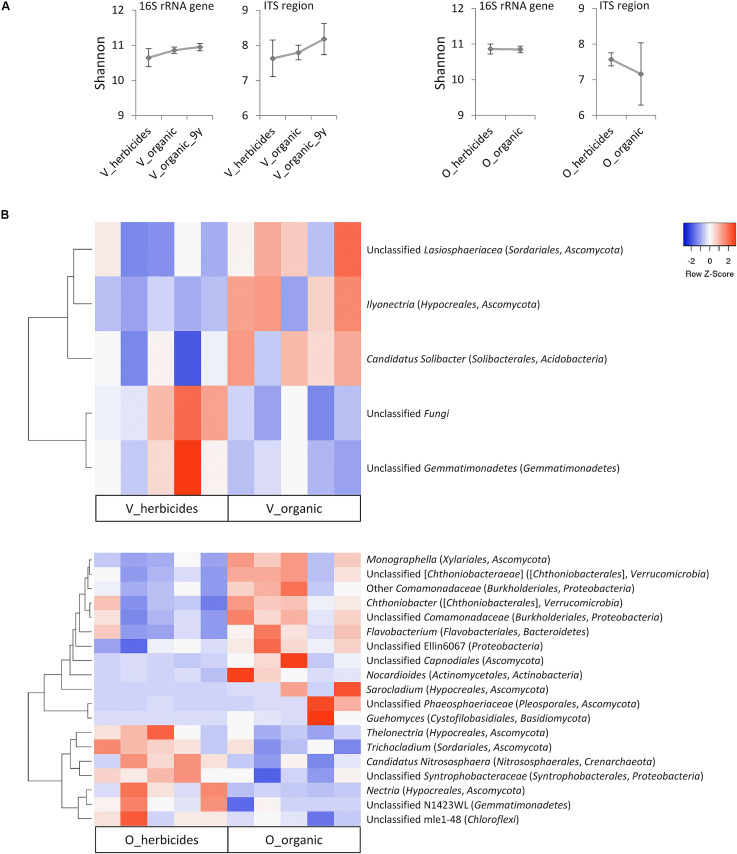
Impact of herbicide application on the soil microbiome of vineyards (V) and orchards (O) in Styria/Austria. **(A)** Alpha diversity determined by Shannon index. Data are mean values ± confidences. Vineyard soils which were knowingly treated organically since the last 9 years were assessed as separate group (V_organic_9y). **(B)** Heat maps displaying the relative abundance of taxa at genus level (>0.1% of overall abundance) with significant difference (non-parametric *t*-test, *p* ≤ 0.05) between herbicide-treated and organic sites. The dendrograms are based on average linkage clustering and Pearson distances.

The microbial alpha diversity (Shannon index) was not significantly impacted (*p* > 0.05) by recent applications of herbicides (Figure 3A). However, for vineyards, a trend to higher microbial soil diversity after several years of organic management could be observed. In orchards, organic management revealed an overall tendency to a lower diversity within the fungal community, however with high variability within the organic sample group and without statistical significance.

In comparison to vineyards under organic management, herbicide-treated vineyard soils revealed significantly lower (*p* ≤ 0.05) relative abundances of unclassified *Lasiosphaeriaceae* (*Sordariales*, *Ascomycota*), *Ilyonectria* (*Hypocreales*, *Ascomycota*), and *Candidatus Solibacter* (*Solibacterales*, *Acidobacteria*). Conversely, herbicide-treated vineyards showed a significant increase in unclassified *Gemmatimonadetes* (phylum *Gemmatimonadetes*) and a group of unclassified *Fungi* (Figure 3B). Herbicide application had a substantial impact on the microbial community composition in orchard soils: 19 out of 209 taxa (genus level, > 0.1% of overall abundance) were significantly shifted. Among those, 12 taxa showed a significantly higher relative abundance in soils of organically managed sites, while for seven taxa the relative abundance was higher in herbicide-treated orchards (Figure 3B).

The herbicide-related shifts at taxonomic level were further verified by analyzing the trophic modes of the fungal community (Supplementary Figure 1). Thereby, no statistically significant shifts (*p* > 0.05) were observed, neither for the mycobiome of vineyard soils nor for orchard soils. However, organically managed orchards showed a trend, although not significant, to a higher abundance of patotrophic-saphrotrophic fungi (2 versus 11% in herbicide-treated orchard soils; *p* = 0.1). The trophic modes of the fungal microbiome inhabiting vineyard soils revealed high stability to herbicide application and remained stable also after 9 years of organic management (maximum ± 2%). Overall, the fraction of pathotrophic fungi was found to be higher in organically managed soils, for both vineyards and orchards (V: 8 vs. 5%, O: 15 vs. 6%). Just for the soils of orchards, the inferred functional composition of the prokaryotic microbiome did show significant differences (*p* ≤ 0.05) in relation to herbicide application, whereby 10 predicted functional categories (KEGG orthology level 3) revealed higher presence in organic soils and only four in herbicide-treated ones (Supplementary Figure 2).

## Discussion

The analysis of the soil microbiota of Styrian agricultural soils revealed an outstanding high bacterial and fungal diversity. Today we know that microbial diversity is a key factor for soil health and in preventing diseases (van Elsas et al., 2012; Berg et al., 2017). Agricultural management as well as distinct soil parameters were identified as drivers of the indigenous microbial communities of Styrian soils, whereby the prokaryotic and fungal community responded differently, and responses differed in orchard and vineyard soils. A detailed understanding of these community responses and interactions can provide the basis to enable the management of vineyards and orchards in a more environmentally friendly and resource-saving way.

Comparative analyses of microbial profiling of prokaryotic 16S rRNA gene fragments and fungal ITS regions revealed several common responses regarding the microbial community composition across all investigated sample groups, especially at higher taxonomic levels. *Proteobacteria* was the most dominant bacterial phylum in Styrian vineyards, orchards, and other agriculturally used soils. This was previously also shown for agricultural soils of the Mediterranean region (Bevivino et al., 2014). Likewise, Janssen (2006) reported *Proteobacteria* to make up the majority (on average 39%) of libraries derived from soil bacterial communities with diverse origin, among those – in accordance with the present study – also *Alphaproteobacteria* were identified as the most dominant. *Alphaproteobacteria* harbor several bacterial lineages that are common plant-associated microorganisms and contribute to plant health and productivity (Wasai and Minamisawa, 2018). Agricultural soils are an important reservoir for these microorganisms that can be attracted and enriched in the rhizosphere of cultivated plants (Sessitsch et al., 2002). *Acidobacteria* was identified as the second highest abundant bacterial phylum in Styrian vineyard and orchard soils. This is as well in accordance with other studies, where *Acidobacteria* were reported to own an average share of 20% of soil bacteria (Janssen, 2006; Naether et al., 2012). Third most represented in the present study was the bacterial phylum *Verrucomicrobia*, which is known as dominant phylum in many soils across the globe (Bergmann et al., 2011). In contrary to the results of Janssen (2006), *Verrucomicrobia* outnumber the abundance of *Actinobacteria* in Styrian vineyard and orchard soils. *Ascomycota* represented the most dominant fungal phylum over all samples, followed by *Zygomycota*, and *Basidiomycota*. Klaubauf et al. (2010) showed similar result for agricultural soils from Lower Austria, whereby in their study the presence of *Ascomycota* was remarkably higher (∼80%).

Vineyards revealed a significantly higher diversity of soil fungi over orchards and other agricultural soils, whereas the prokaryotic diversity was unaffected by soil usage. Also, the community structure differed significantly between vineyard and orchard mycobiomes, while there was a negligible effect for prokaryotes. The most distinct difference was a significantly higher abundance of the fungal class *Tremellomycetes* in orchard soils. This was due to the occurrence of the genus *Solicoccozyma*, a basidiomycetous yeast commonly found in soils with high salt content (Mokhtarnejad et al., 2016; Zajc et al., 2017). Although all investigated soils were classified as non-saline, orchard soils revealed on average higher salinity (mean EC = 0.40 mS cm^–1^) than vineyards (mean EC = 0.28 mS cm^–1^) and other agricultural soils (mean EC = 0.25 mS cm^–1^). This is most probably a result of the fertilization management; application rates in orchards are generally higher.

The acid-base balance, disorders in the sorption complex, content and quality of organic substance as well as individual nutrients (such as zinc and manganese) were identified as important drivers of the microbial community structure of Styrian vineyard and orchard soils. Soil pH was one of the most important edaphic modulators in both, vineyard and orchard soils. In a continental-scale study, Fierer and Jackson (2006) found that diversity and richness of soil bacterial communities was strongly correlated with soil pH. Bacterial diversity was highest in neutral soils and lower in acidic soils (Fierer and Jackson, 2006). Similarly, Wu et al. (2017) reported that bacterial diversity in arable soils is strongly related to soil pH, with lower diversity under acidic and higher diversity under neutral pH conditions. On an experimental farm maintaining a pH gradient from 4.5 to 7.5, shifts were observed even at higher taxonomic levels (phylum, class) across the gradient (Bartram et al., 2014). The pH values (in water) from soils of vineyards ranged from 5.5 to 7.9. Soils of orchards had a slightly narrower pH range from 5.9 to 7.6. In the same environment, soil fungi commonly show a growth optimum of one to two pH units lower than bacteria (Piña and Cervantes, 1996), where soil pH causes a physiological limitation on fungal survival and growth (Zhang et al., 2016). Both in vineyards and orchards, the acid-base ratio and the sorption complex played a major role in shaping microbial communities. The relationship to the quantity and quality of organic matter seems to be stronger within the orchards than in vineyards. Individual sampling sites with a combination of different suboptimal edaphic factors showed a particularly strong effect on microbial diversity and composition, this could be referred to as soil fatigue.

The impact of herbicide application on the microbial soil community was assessed based on the sites’ treatment history. Soils of vineyards and orchards were analyzed based on the information if they were treated with herbicides during the last two years or managed organically, independent from herbicide type, application form and period. In comparison to orchards, the microbiome of vineyard soils maintained a higher stability in terms of taxonomy and inferred functionality when herbicides were applied. In contrast to vineyards, where the application of herbicides tends to be associated with a general reduction in microbial soil diversity, orchard soils exhibited drastic shifts within community composition at taxonomic and predicted functional level. For example, in orchards, herbicides seem to have a substantial impact on the bacterial order *Chthoniobacterales* (genus *Chthoniobacter* and others), with a decreased abundance in herbicide-treated soils. *Chthoniobacterales* are rod-shaped or pleomorphic cells, which are common in soil but not well-studied so far (Sangwan et al., 2004). Hirsch et al. (2017) described them as rapid responders to soil management changes, with highest abundance in grassland, followed by arable soil, and least in bare fallow soil. According to genomic data, *Spartobacteria* (*Chthoniobacterales*) contribute to the cycling of carbon by the degradation of various complex carbohydrates, such as cellulose and xylan (Herlemann et al., 2013). In the microbiome of lichens, transcriptomics suggest their involvement in the metabolism of aromatic compounds (degradation of phenolic substances), production of various vitamins, and defense against antibiotics (fluoroquinolones) and oxidative stress (Cernava et al., 2017). Other identified genera with increased abundance in herbicide-free orchard soils comprise potential plant growth promoters and antagonists of phytopathogens, including *Flavobacterium* (Soltani et al., 2010) and *Monographella* (Berg et al., 2005). Moreover, soils of herbicide-treated orchards revealed a significantly higher presence of the potential apple pathogenic fungus *Nectria*, the causal agent of apple canker, and also of the closely related genus *Thelonectria*. Contrarily, vineyards revealed higher abundances of the potentially pathogenic genus *Ilyonectria* (black-foot disease) in organic soils. It was also evident from our data that a lack of greening in the plant strip through the regular use of herbicides led to humus degradation over years. A perceived reduction in fruit quality and yield can potentially lead to anthropogenic over-fertilization. However, this has generally negative effects by inducing additional acidification. That is in turn linked to a decrease in available carbon (Högberg et al., 2007) and microbial, in particular bacterial, diversity (Fierer and Jackson, 2006; Wu et al., 2017) – a downward spiral of which it is difficult to re-escape.

In the end, the limitations of marker gene-based approaches, as applied in the present study, should be briefly discussed. In particularly mentioned should be a possible bias toward certain microbial groups incorporated with the initial amplification step (Caporaso et al., 2012; Klindworth et al., 2013). In order to minimize this bias and for comparability reasons, the protocol applied in the present study was following the latest guidelines recommended by the Earth Microbiome Project (Gilbert et al., 2014, 2018). A critical point in the analysis of fungi is that ITS regions can vary drastically in length for different taxa. In some cases they may be longer than the possible merged read length of paired-end reads and will be discarded in the data analysis workflow (Hoggard et al., 2018). To rule this out as far as possible, in the present study, longest possible paired-end reads were sequenced (2 × 300 bp). However, also this length can far be exceeded by the ITS1 region of some taxa. Therefore, an additional comparison of the data using merged paired-end reads vs. analyzing only forward reads was performed, whereby a momentous bias resulting from exceeding the ITS1 read length could be excluded (Supplementary Table 1). In general, microbial profiling based on rRNA genes or ITS regions is limited to measurements of taxonomy and diversity and allows no direct inference to the metabolic potential of a microbial community (White et al., 2017). Tools like PICRUSt (Langille et al., 2013) predict functional profiles of microbial communities by linking marker genes with the nearest organism for which a whole genome sequence is available. This can be problematic, especially when studying microbiomes containing large proportions of not well-characterized taxa. FUNGuild (Nguyen et al., 2016) classifies fungal taxa by their probable ecological guild. An important caution about the accuracy of this assignment is the fact that some fungi do not fall exclusively into a single guild. Taxonomy-based functional predictions should therefore be interpreted cautiously.

The overall findings of our large-scale study indicate that certain agricultural management practices as well as distinct soil parameters have a substantial effect on indigenous microbial communities in agricultural soils. Moreover, we could show that responses to distinct parameters differed in orchards and vineyards as well as that bacterial and fungal community showed different responses to the same abiotic factor. These findings provide the basis to adapt soil management practices in the future in order to maintain a healthy microbiome in agricultural soils.

## Data Availability Statement

The datasets generated and analyzed for this study can be found in the European Nucleotide Archive (www.ebi.ac.uk/ena) under the BioProject accession number PRJEB36740.

## Author Contributions

RM (sampling design), MK, and GB (experimental design) conceived and designed the study. MK, PW, HM, and HU analyzed the data. GB, RM, and HU contributed reagents, materials, and analysis tools. MK, PW, TC, and GB wrote the manuscript.

## Conflict of Interest

HU is founder and CEO of the company TB Unterfrauner GmbH. The remaining authors declare that the research was conducted in the absence of any commercial or financial relationships that could be construed as a potential conflict of interest.

## Footnotes

1 https://www.statistik.at

2 http://press.igsb.anl.gov/earthmicrobiome/protocols-and-standards

## References

[B1] Ahmed R. A. El-Shehawy M. A. Lutang L. (2011). The structure and competitiveness of China’s apple exports. *World J. Agric. Sci.* 7 678–683.

[B2] Babicki S. Arndt D. Marcu A. Liang Y. Grant J. R. Maciejewski A. (2016). Heatmapper: web-enabled heat mapping for all. *Nucleic Acids Res.* 44 W147–W153. 10.1093/nar/gkw419 27190236 PMC4987948

[B3] Bardgett R. D. van der Putten W. H. (2014). Belowground biodiversity and ecosystem functioning. *Nature* 515 505–511. 25428498 10.1038/nature13855

[B4] Bartram A. K. Jiang X. Lynch M. D. Masella A. P. Nicol G. W. Dushoff J. (2014). Exploring links between pH and bacterial community composition in soils from the craibstone experimental farm. *FEMS Microbiol. Ecol.* 87 403–415. 10.1111/1574-6941.12231 24117982

[B5] Berg G. Köberl M. Rybakova D. Müller H. Grosch R. Smalla K. (2017). Plant microbial diversity is suggested as the key to future biocontrol and health trends. *FEMS Microbiol. Ecol.* 93:fix050 10.1093/femsec/fix050 28430944

[B6] Berg G. Smalla K. (2009). Plant species and soil type cooperatively shape the structure and function of microbial communities in the rhizosphere. *FEMS Microbiol. Ecol.* 68 1–13. 10.1111/j.1574-6941.2009.00654.x 19243436

[B7] Berg G. Zachow C. Lottmann J. Götz M. Costa R. Smalla K. (2005). Impact of plant species and site on rhizosphere-associated fungi antagonistic to *Verticillium dahliae* Kleb. *Appl. Environ. Microbiol.* 71 4203–4213. 10.1128/AEM.71.8.4203-4213.2005 16085804 PMC1183293

[B8] Bergmann G. T. Bates S. T. Eilers K. G. Lauber C. L. Caporaso J. G. Walters W. A. (2011). The under-recognized dominance of *Verrucomicrobia* in soil bacterial communities. *Soil Biol. Biochem.* 43 1450–1455. 10.1016/j.soilbio.2011.03.012 22267877 PMC3260529

[B9] Berlanas C. Berbegal M. Elena G. Laidani M. Cibriain J. F. Sagües A. (2019). The fungal and bacterial rhizosphere microbiome associated with grapevine rootstock genotypes in mature and young vineyards. *Front. Microbiol.* 10:1142 10.3389/fmicb.2019.01142 PMC6538693 31178845

[B10] Bevivino A. Paganin P. Bacci G. Florio A. Pellicer M. S. Papaleo M. C. (2014). Soil bacterial community response to differences in agricultural management along with seasonal changes in a Mediterranean region. *PLoS One* 9:e105515 10.1371/journal.pone.0105515 PMC4140800 25144665

[B11] Caporaso J. G. Bittinger K. Bushman F. D. DeSantis T. Z. Andersen G. L. Knight R. (2010a). PyNAST: a flexible tool for aligning sequences to a template alignment. *Bioinformatics* 26 266–267. 10.1093/bioinformatics/btp636 19914921 PMC2804299

[B12] Caporaso J. G. Kuczynski J. Stombaugh J. Bittinger K. Bushman F. D. Costello E. K. (2010b). QIIME allows analysis of high-throughput community sequencing data. *Nat. Methods* 7 335–336. 10.1038/nmeth.f.303 20383131 PMC3156573

[B13] Caporaso J. G. Lauber C. L. Walters W. A. Berg-Lyons D. Huntley J. Fierer N. (2012). Ultra-high-throughput microbial community analysis on the Illumina HiSeq and MiSeq platforms. *ISME J.* 6 1621–1624. 10.1038/ismej.2012.8 22402401 PMC3400413

[B14] Cernava T. Erlacher A. Aschenbrenner I. A. Krug L. Lassek C. Riedel K. (2017). Deciphering functional diversification within the lichen microbiota by meta-omics. *Microbiome* 5:82 10.1186/s40168-017-0303-5 PMC5518139 28724401

[B15] Coller E. Cestaro A. Zanzotti R. Bertoldi D. Pindo M. Larger S. (2019). Microbiome of vineyard soils is shaped by geography and management. *Microbiome* 7:140 10.1186/s40168-019-0758-7 PMC6839268 31699155

[B16] Delgado-Baquerizo M. Oliverio A. M. Brewer T. E. Benavent-González A. Eldridge D. J. Bardgett R. D. (2018). A global atlas of the dominant bacteria found in soil. *Science* 359 320–325. 10.1126/science.aap9516 29348236

[B17] Eccher G. Ferrero S. Populin F. Colombo L. Botton A. (2014). Apple (*Malus domestica* L. Borkh) as an emerging model for fruit development. *Plant Biosyst.* 148 157–168.

[B18] Edgar R. C. (2010). Search and clustering orders of magnitude faster than BLAST. *Bioinformatics* 26 2460–2461. 10.1093/bioinformatics/btq461 20709691

[B19] FAO, and ITPS (2015). *Status of the World’s Soil Resources (SWSR) – Main Report. Food and Agriculture Organization of the United Nations and Intergovernmental Technical Panel on Soils.* Rome: FAO.

[B20] FAOSTAT (2016). Statistical Database of the Food and Agriculture Organization of the United Nations. Available: http://www.fao.org/faostat/en/#data/TP (accessed November 19, 2019).

[B21] FAOSTAT (2017). *Statistical Database of the Food and Agriculture Organization of the United Nations.* Available: http://www.fao.org/faostat/en/#data/QC (accessed November 19, 2019).

[B22] Fierer N. (2017). Embracing the unknown: disentangling the complexities of the soil microbiome. *Nat. Rev. Microbiol.* 15 579–590. 10.1038/nrmicro.2017.87 28824177

[B23] Fierer N. Jackson R. B. (2006). The diversity and biogeography of soil bacterial communities. *Proc. Natl. Acad. Sci. U.S.A.* 103 626–631. 10.1073/pnas.0507535103 16407148 PMC1334650

[B24] Fra̧c M. Hannula S. E. Bełka M. Jȩdryczka M. (2018). Fungal biodiversity and their role in soil health. *Front. Microbiol.* 9:707 10.3389/fmicb.2019.0707 PMC5932366 29755421

[B25] Gagelidze N. A. Amiranashvili L. L. Sadunishvili T. A. Kvesitadze G. I. Urushadze T. F. Kvrivishvili T. O. (2018). Bacterial composition of different types of soils of Georgia. *Ann. Agrar. Sci.* 16 17–21.

[B26] Gardes M. Bruns T. D. (1993). ITS primers with enhanced specificity for basidiomycetes – application to the identification of mycorrhizae and rusts. *Mol. Ecol.* 2 113–118. 10.1111/j.1365-294x.1993.tb00005.x 8180733

[B27] Gilbert J. A. Jansson J. K. Knight R. (2014). The Earth Microbiome Project: successes and aspirations. *BMC Biol.* 12:69 10.1186/s12915-014-0069-1 PMC4141107 25184604

[B28] Gilbert J. A. Jansson J. K. Knight R. (2018). Earth Microbiome Project and global systems biology. *mSystems* 3:e0217-17 10.1128/mSystems.00217-17 PMC5893859 29657969

[B29] Herlemann D. P. Lundin D. Labrenz M. Jürgens K. Zheng Z. Aspeborg H. (2013). Metagenomic *de novo* assembly of an aquatic representative of the verrucomicrobial class *Spartobacteria*. *mBio* 4:e0569-12 10.1128/mBio.00569-12 PMC3663571 23716574

[B30] Hirsch P. R. Jhurreea D. Williams J. K. Murray P. J. Scott T. Misselbrook T. H. (2017). Soil resilience and recovery: rapid community responses to management changes. *Plant Soil* 412 283–297. 10.1007/s11104-016-3068-x 32165771 PMC7045894

[B31] Högberg M. N. Högberg P. Myrold D. D. (2007). Is microbial community composition in boreal forest soils determined by pH, C-to-N ratio, the trees, or all three? *Oecologia* 150 590–601. 10.1007/s00442-006-0562-5 17033802

[B32] Hoggard M. Vesty A. Wong G. Montgomery J. M. Fourie C. Douglas R. G. (2018). Characterizing the human mycobiota: a comparison of small subunit rRNA, ITS1, ITS2, and large subunit rRNA genomic targets. *Front. Microbiol.* 9:2208 10.3389/fmicb.2019.02208 PMC6157398 30283425

[B33] International Organisation of Vine and Wine (2016). *World Vitiviniculture Situation – OIV Statistical Report on World Vitiviniculture.* Available: http://www.oiv.int/public/medias/5029/world-vitiviniculture-situation-2016.pdf (accessed November 19, 2019).

[B34] Janssen P. H. (2006). Identifying the dominant soil bacterial taxa in libraries of 16S rRNA and 16S rRNA genes. *Appl. Environ. Microbiol.* 72 1719–1728. 10.1128/AEM.72.3.1719-1728.2006 16517615 PMC1393246

[B35] Jansson J. K. (2011). Towards “Tera-Terra”: terabase sequencing of terrestrial metagenomes. *Microbe* 6 309–315.

[B36] Jansson J. K. Hofmockel K. S. (2018). The soil microbiome – from metagenomics to metaphenomics. *Curr. Opin. Microbiol.* 43 162–168. 29454931 10.1016/j.mib.2018.01.013

[B37] Klaubauf S. Inselsbacher E. Zechmeister-Boltenstern S. Wanek W. Gottsberger R. Strauss J. (2010). Molecular diversity of fungal communities in agricultural soils from Lower Austria. *Fungal Divers.* 44 65–75. 10.1007/s13225-010-0053-1 23794962 PMC3688302

[B38] Klindworth A. Pruesse E. Schweer T. Peplies J. Quast C. Horn M. (2013). Evaluation of general 16S ribosomal RNA gene PCR primers for classical and next-generation sequencing-based diversity studies. *Nucleic Acids Res.* 41:e1 10.1093/nar/gks808 PMC3592464 22933715

[B39] Langille M. G. Zaneveld J. Caporaso J. G. McDonald D. Knights D. Reyes J. A. (2013). Predictive functional profiling of microbial communities using 16S rRNA marker gene sequences. *Nat. Biotechnol.* 31 814–821. 10.1038/nbt.2676 23975157 PMC3819121

[B40] Liu J. Abdelfattah A. Norelli J. Burchard E. Schena L. Droby S. (2018). Apple endophytic microbiota of different rootstock/scion combinations suggests a genotype-specific influence. *Microbiome* 6:18 10.1186/s40168-018-0403-x PMC5787276 29374490

[B41] Marasco R. Rolli E. Fusi M. Michoud G. Daffonchio D. (2018). Grapevine rootstocks shape underground bacterial microbiome and networking but not potential functionality. *Microbiome* 6:3 10.1186/s40168-017-0391-2 PMC5751889 29298729

[B42] Mazzola M. Hewavitharana S. S. Strauss S. L. (2015). *Brassica* seed meal soil amendments transform the rhizosphere microbiome and improve apple production through resistance to pathogen reinfestation. *Phytopathology* 105 460–469. 10.1094/PHYTO-09-14-0247-R 25412009

[B43] Mokhtarnejad L. Arzanlou M. Babai-Ahari A. Di Mauro S. Onofri A. Buzzini P. (2016). Characterization of basidiomycetous yeasts in hypersaline soils of the Urmia Lake National Park. Iran. *Extremophiles* 20 915–928. 10.1007/s00792-016-0883-1 27770301

[B44] Naether A. Foesel B. U. Naegele V. Wüst P. K. Weinert J. Bonkowski M. (2012). Environmental factors affect acidobacterial communities below the subgroup level in grassland and forest soils. *Appl. Environ. Microbiol.* 78 7398–7406. 10.1128/AEM.01325-12 22885760 PMC3457104

[B45] Nguyen N. H. Song Z. Bates S. T. Branco S. Tedersoo L. Menke J. (2016). FUNGuild: an open annotation tool for parsing fungal community datasets by ecological guild. *Fungal Ecol.* 20 241–248.

[B46] Parada A. E. Needham D. M. Fuhrman J. A. (2016). Every base matters: assessing small subunit rRNA primers for marine microbiomes with mock communities, time series and global field samples. *Environ. Microbiol.* 18 1403–1414. 10.1111/1462-2920.13023 26271760

[B47] Parks D. H. Tyson G. W. Hugenholtz P. Beiko R. G. (2014). STAMP: statistical analysis of taxonomic and functional profiles. *Bioinformatics* 30 3123–3124. 10.1093/bioinformatics/btu494 25061070 PMC4609014

[B48] Piña R. G. Cervantes C. (1996). Microbial interactions with aluminium. *Biometals* 9 311–316. 10.1007/BF00817932 8696081

[B49] Prosser J. I. (2015). Dispersing misconceptions and identifying opportunities for the use of ‘omics’ in soil microbial ecology. *Nat. Rev. Microbiol.* 13 439–446. 10.1038/nrmicro3468 26052662

[B50] Quince C. Lanzen A. Davenport R. J. Turnbaugh P. J. (2011). Removing noise from pyrosequenced amplicons. *BMC Bioinform.* 12:38. 10.1186/1471-2105-12-38 PMC3045300 21276213

[B51] Sangwan P. Chen X. Hugenholtz P. Janssen P. H. (2004). Chthoniobacter flavus gen. nov., sp. nov., the first pure-culture representative of subdivision two, *Spartobacteria* classis nov., of the phylum *Verrucomicrobia*. *Appl. Environ. Microbiol.* 70 5875–5881. 10.1128/AEM.70.10.5875-5881.2004 15466527 PMC522106

[B52] Santoyo G. Hernández-Pacheco C. Hernández-Salmerón J. Hernández-León R. (2017). The role of abiotic factors modulating the plant-microbe-soil interactions: toward sustainable agriculture – a review. *Span J. Agric. Res.* 15:e03R01.

[B53] Sessitsch A. Howieson J. G. Perret X. Antoun H. Martínez-Romero E. (2002). Advances in *Rhizobium* research. *Crit. Rev. Plant Sci.* 21 323–378.

[B54] Soltani A. Khavazi K. Asadi-Rahmani H. Omidvari M. Dahaji P. A. Mirhoseyni H. (2010). Plant growth promoting characteristics in some *Flavobacterium* spp. isolated from soils of Iran. *J. Agric. Sci.* 2 106–115.

[B55] van Elsas J. D. Chiurazzi M. Mallon C. A. Elhottova D. Kristufek V. Salles J. F. (2012). Microbial diversity determines the invasion of soil by a bacterial pathogen. *Proc. Natl. Acad. Sci. U.S.A.* 109 1159–1164. 10.1073/pnas.1109326109 22232669 PMC3268289

[B56] Wasai S. Minamisawa K. (2018). Plant-associated microbes: from rhizobia to plant microbiomes. *Microb. Environ.* 33 1–3. 10.1264/jsme2.ME3301rh PMC5877334 29593170

[B57] White R. A. III Rivas-Ubach A. Borkum M. I. Köberl M. Bilbao A. Colby S. M. (2017). The state of rhizospheric science in the era of multi-omics: a practical guide to omics technologies. *Rhizosphere* 3 212–221.

[B58] White T. J. Bruns T. Lee S. Taylor J. W. (1990). “Amplification and direct sequencing of fungal ribosomal RNA genes for phylogenetics,” in *PCR Protocols: A Guide To Methods And Applications*, eds Innis M. A. Gelfand D. H. Sninsky J. J. White T. J. (New York, NY: Academic Press), 315–322.

[B59] Wilpiszeski R. L. Aufrecht J. A. Retterer S. T. Sullivan M. B. Graham D. E. Pierce E. M. (2019). Soil aggregate microbial communities: towards understanding microbiome interactions at biologically relevant scales. *Appl. Environ. Microbiol.* 85:e0324-19 10.1128/AEM.00324-19 PMC6606860 31076430

[B60] Wu Y. Zeng J. Zhu Q. Zhang Z. Lin X. (2017). pH is the primary determinant of the bacterial community structure in agricultural soils impacted by polycyclic aromatic hydrocarbon pollution. *Sci. Rep.* 7:40093 10.1038/srep40093 PMC5209717 28051171

[B61] Zajc J. Zalar P. Gunde-Cimerman N. (2017). “Yeasts in hypersaline habitats,” in *Yeasts in Natural Ecosystems: Diversity*, eds Buzzini P. Lachance M. A. Yurkov A. (Berlin: Springer), 293–329.

[B62] Zhang Q. Sun J. Liu S. Wei Q. (2013). Manure refinement affects apple rhizosphere bacterial community structure: a study in sandy soil. *PLoS One* 8:e76937 10.1371/journal.pone.076937 PMC3796563 24155909

[B63] Zhang T. Wang N. F. Liu H. Y. Zhang Y. Q. Yu L. Y. (2016). Soil pH is a key determinant of soil fungal community composition in the Ny-Ålesund Region, Svalbard (High Arctic). *Front. Microbiol.* 7:227 10.3389/fmicb.2019.0227 PMC4767930 26955371

