# Supplementary material for: Unraveling the Complexity of Soil Microbiomes in a Large-Scale Study Subjected to Different Agricultural Management in Styria
Source: Front Microbiol. 2020 May 25;11:1052. doi: 10.3389/fmicb.2020.01052 (PMC7261914; doi:10.3389/fmicb.2020.01052)
Supplement: Supplementary file 1 [file Data_Sheet_1.PDF]

Supplementary Material

Supplementary Table 1. OTU counts and relative abundances of fungal phyla (in bold) and classes with >1% of overall relative abundance expressed as mean values ± confidences based on merged paired-end ITS1 reads processing vs. analyzing only forward reads.

		**merged pipeline**	**forward reads only**
**OTUs**		**53,710 OTUs**	**69,801 OTUs**
Ascomycota		**44.6 ± 1.7%**	**53.3 ± 1.9%**
	Sordariomycetes	26.0 ± 1.4%	24.1 ± 1.4%
	Dothideomycetes	6.9 ± 0.9%	14.3 ± 1.1%
	Leotiomycetes	4.2 ± 0.6%	4.5 ± 0.9%
	Eurotiomycetes	1.4 ± 0.2%	2.4 ± 0.3%
	unidentified *Ascomycota*	4.4 ± 0.4%	4.3 ± 0.5%
Zygomycota		**25.7 ± 2.0%**	**24.6 ± 1.8%**
	*Mortierellomycotina*_cls_*Incertae_sedis*	25.5 ± 2.0%	24.6 ± 1.8%
Basidiomycota		**10.9 ± 1.4%**	**13.3 ± 1.7%**
	Tremellomycetes	6.7 ± 1.3%	10.2 ± 1.7%
	Agaricomycetes	3.9 ± 0.9%	2.9 ± 0.7%
**unidentified *Fungi***		**17.0 ± 1.2%**	**6.2 ± 0.5%**
	unidentified *Fungi*	17.0 ± 1.2%	6.2 ± 0.5%

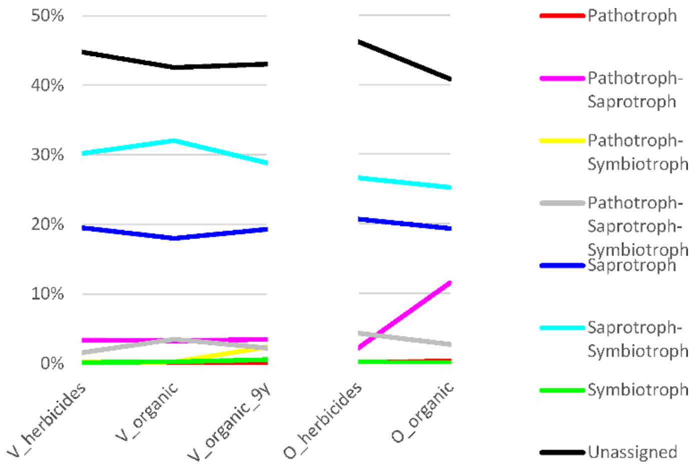

Supplementary Figure 1. Impact of herbicide application on the trophic modes of the fungal communities inhabiting vineyard (V) and orchard (O) soils in Styria/Austria. Vineyard soils which were knowingly treated organically since the last nine years were assessed as separate group (V_organic_9y).

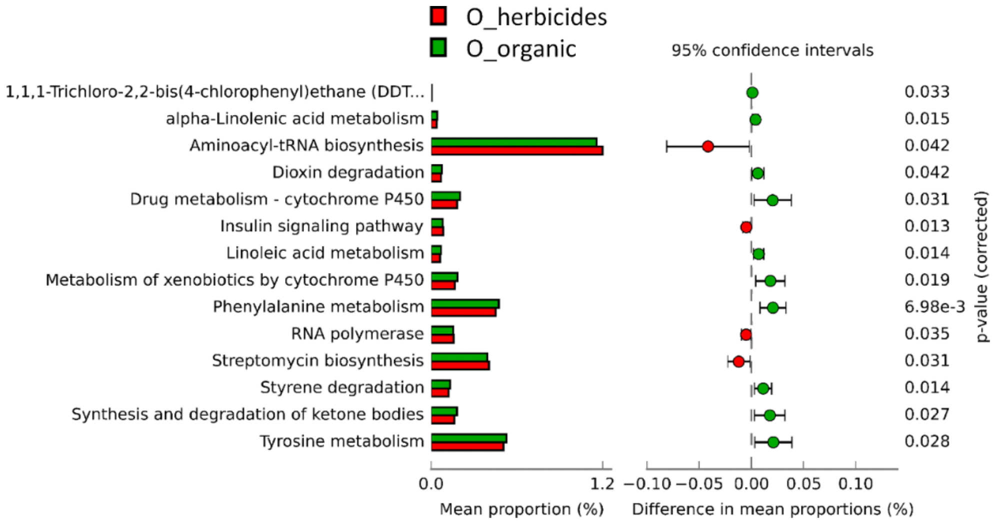

Supplementary Figure 2. Inferred KEGG orthology (level 3) annotations of the prokaryotic communities with significant difference (two-sided Welch's *t*-test, $p \leq 0.05$) between herbicide-treated and organic orchards (O).
